# In depth analysis of the association of FTO SNP (rs9939609) with the expression of classical phenotype of PCOS: a Sri Lankan study

**DOI:** 10.1186/s12881-020-0961-1

**Published:** 2020-02-12

**Authors:** Umayal Branavan, Sulochana Wijesundera, Vishvanath Chandrasekaran, Carukshi Arambepola, Chandrika Wijeyaratne

**Affiliations:** 1 Department of Obstetrics and Gynecology, Faculty of Medicine, University of Colombo, PO Box 271, Kynsey Road, Colombo, 08 Sri Lanka; 2 Department of Biochemistry and Molecular Biology, Faculty of Medicine, University of Colombo, PO Box 271, Kynsey Road, Colombo, 08 Sri Lanka; 3 Department of Chemistry, Faculty of Science, University of Colombo, Colombo, 07 Sri Lanka; 4 Department of Community Medicine, Faculty of Medicine, University of Colombo, PO Box 271, Kynsey Road, Colombo, 08 Sri Lanka

**Keywords:** Polycystic ovary syndrome (PCOS), FTO SNP rs9939609, Sri Lankan

## Abstract

**Background:**

PCOS is a common disorder of women due to genetic, endocrine and environmental effects that manifests from puberty. The rs9939609 variant of fat mass and obesity associated (FTO) gene is linked to metabolic derangement in PCOS. We previously identified FTO (rs9939609) as a susceptibility locus for PCOS among Sri Lankan women and also explored the role of kisspeptin. Associated factors of the FTO candidate gene among South Asians with PCOS are unknown.

**Methods:**

This study aimed to determine the association between FTO (rs9939609) polymorphism with clinical (BMI, acanthosis nigricans, hirsutism) and biochemical (serum kisspeptin and testosterone levels) characteristics of PCOS in a cohort of Sri Lankan women. Genetic and clinical data including serum kisspeptin and testosterone concentrations of our previously reported cases (*n* = 55) and controls (*n* = 110) were re-analyzed, specifically for an association with rs9939609 variant of FTO gene.

**Results:**

Logistic regression analysis (AA – OR = 5.7, 95% CI = 2.41–13.63, *p* < 0.05) and genetic inheritance analysis (AA – OR = 5.49, 95%CI = 2.34–12.88, *p* < 0.05) showed that FTO (rs9939609) polymorphism is significantly associated with PCOS and its metabolic manifestations. Serum testosterone was significantly higher in affected women with mutant genotypes (AA+AT) than with the normal allele (TT) (*p* < 0.05). Although serum kisspeptin was higher in subjects with PCOS and mutant alleles than controls, this difference was not significant (*p* > 0.05).

**Conclusion:**

FTO gene variant rs9939609 is associated with hyperandrogenemia and metabolic manifestations of PCOS among women of Sri Lankan descent with the well-characterized phenotype. Serum kisspeptin and the FTO genotypes lack a significant association when adjusted for confounders.

## Background

Polycystic ovary syndrome is the commonest hormonal derangement among young women, from early reproductive years [1]. The well characterized phenotype of PCOS is commonly associated with insulin resistance, obesity and future metabolic/cardiovascular outcomes such as diabetes mellitus, hypertension, carotid artery intimal thickening, and pregnancy related metabolic risks [2–6]. South Asians have a greater expression of the metabolic manifestations of PCOS, occurring from a younger age and with a greater degree of insulin resistance, linked to central adiposity, as opposed to white Caucasians with the condition [2].

The etiology of PCOS is undetermined. It is viewed as a complex trait due to heritable and environmental factors [7–9]. In 2007, genome-wide association study identified the fat mass and obesity-associated (FTO) gene as an obesity susceptibility gene [10]. The human FTO gene is located on chromosome 16 and expressed in a wide range of tissues, including adipose tissue and specific areas of brain and muscles, suggesting its potential role in body weight regulation [11]. Several single nucleotide polymorphisms (SNPs) of the FTO gene have been described. The variant FTO rs9939609 is the most extensively studied, located within the first FTO intron which has two alleles, A and T, the former linked to an increased risk for both obesity and type 2 diabetes mellitus [12].

The hypothalamic pituitary gonadal (HPG) axis plays a central role in reproduction. Important regulators of puberty include kisspeptin, a peptide product of Kiss1 gene, and its receptor GPR54 [13]. Activation of GnRH neurons is critical for the onset of puberty [14]. GPR54 receptor being located on neurons that secrete GnRH implies an important role for kisspeptin/GPR54 signaling in reproduction [15, 16].

The FTO gene affects adiposity, insulin resistance and the development of type 2 diabetes mellitus. Obesity and PCOS are closely related [17, 18]. The FTO gene variants have been demonstrated to be a key risk for PCOS [5, 19–21]. Since PCOS, in its well characterized form, often begins from adolescence, especially among South Asians [22], it is reasonable to hypothesize that FTO rs9939609 polymorphism influences the Kiss1/kisspeptin/GPR54 pathway in PCOS. Furthermore, while FTO variants are linked to insulin resistance and glucose intolerance in PCOS [23], there is paucity of literature on the relationship between FTO variants and hyperandrogenaemia [11, 24].

We aimed to identify any association of FTO gene variant rs9939609 with clinical (metabolic and hyperandrogenic) and hormonal (serum kisspeptin and testosterone) characteristics of PCOS among young Sri Lankan women with well characterized PCOS.

## Methods

This study was carried out at the Endocrine Clinic of the University Unit, Colombo, Sri Lanka, during the period from 1st January 2016 to 31st December 2016. Ethical approval for this study was obtained from the Ethics Review Committee, Faculty of Medicine, University of Colombo.

Diagnosis of PCOS was based on the Rotterdam criteria [25]. Details of sample size calculation [26], inclusion and exclusion criteria of study subjects and control group are described in previous reports [27–29] are given below:

### Sample size calculation

The sample size was calculated based on Schlesselman case control study formula [26];

$$ \mathrm{n}=\frac{{\left({z}_{\alpha}\sqrt{2\overline{p}\overline{q\ }\ }\kern0.75em +\kern0.5em {z}_{\beta }\ \sqrt{p_1{q}_1+{p}_0{q}_0}\right)}^2}{{\left({p}_1-{p}_0\right)}^2} $$

The exposure rate of allele frequency of SNPs among controls (30–44%) was based on literature from other countries as no studies have been done in Sri Lanka [10, 30–32]. The estimated proportion of 38% was used in sample size calculation.

The OR used in this study was 2.7; because Sri Lankans with anovulatory PCOS manifest severe symptoms at a younger age, with greater IR and a higher prevalence of metabolic syndrome than white Europeans [2].

P1 = Proportion of exposure among cases was calculated using the following formula**;**

$$ {p}_1=\frac{p_0R}{\left[1+{p}_0\left(R-1\right)\right]}. $$

The total number of cases recruited for this study was 55 and by selecting double the number of controls per cases, the final total number of controls was 110. Thus, in total 165 subjects were included in this study.

### Recruitment of subjects

#### Inclusion criteria

Adolescent women whose symptoms manifested between 11 and 19 years of age (WHO) with all 3 diagnostic criteria present between 16 and 19 years of age [33] were recruited for this study. The lower age limit for inclusion was 15 years, since the mean age of menarche reported among Sri Lankan girls is 13.54 years (standard deviation, 0.86) and 2 years after menarche was required to exclude the period of menstrual irregularity that usually follows menarche [34].

Women with all 3 following diagnostic criteria were recruited as cases: oligomenorrhoea/oligo-ovulation, clinical or biochemical hyperandrogenism and polycystic ovaries on ultrasound. Details of diagnostic criteria were described briefly in our previous studies [27–29].

Women excluded from the study were those with inherited disorders of IR such as Rabson –Mendenhall syndrome, Cushing syndrome, hyperprolactinaemia, untreated hypothyroidism, congenital adrenal hyperplasia or with an androgen secreting ovarian/adrenal tumour and those taking corticosteroid, antiepileptic or antipsychotic drugs or hormonal contraception and those currently pregnant or in the first postpartum year.

#### Control sample

Working women of reproductive age from similar ethnic and social background as the affected subjects were approached. Consenting, asymptomatic, normo-androgenic and normal cycling since adolescence women in whom PCOS was objectively excluded by clinical, biochemical and ultrasound assessment, were recruited as controls.

### Clinical and biochemical evaluation

Socio demographic, reproductive information (menstruation, fertility), age and degree of severity of clinical feature of PCOS, drug history, family history of diabetes, anthropometry (BMI and central obesity), resting blood pressure, hyperandrogensim assessed by a single clinical observer that includes hirsutism (modified FG score), temporal hair loss, acne, acanthosis nigricans, ovarian ultrasound, serum kisspeptin and testosterone concentrations of all subjects were utilized from our previously reported studies [27–29].

### Statistical analysis

Statistical analysis was carried out with previously reported SNPs of the obesity gene (FTO), selected candidate genes of HPG axis (Kiss1, GPR54, GnRH, FSHB, FSHR, LHB, LHCGR) and insulin receptor gene (INSR) [28, 29] and serum kisspeptin and testosterone concentration [27].

The Kolmogorov-Smirnov test was used to test the normality of distribution. Values with a biological distribution are presented as mean ± standard error for mean. Comparison of means between cases (those with PCOS) and controls (those without PCOS) was performed with independent sample t test. Chi-square test was used for comparison of genotype frequency between groups. It was also used for describing the correlation of genetic alleles with other numeric variables. To assess the magnitude of the risk factors in the development of PCOS, first all cases and controls were compared using crude odds ratio (OR) and 95% confidence interval (CI) by binary logistic regression (forward LR) method. Factors assessed were: BMI, serum kisspeptin and testosterone concentration with the obesity gene - FTO (rs9939609), HPG axis genes – Kiss1 SNPs (rs5780218, rs4889), GPR54 SNPs (rs10407968, rs12507294, rs350131, chr19:918686, chr19:918735), GnRH (rs6185), FSHB (rs6169), FSHR (rs6165/rs6166) and LHCGR (rs2293275) and insulin receptor gene (rs1799817), all of which were included to the logistic regression analysis in order to identify risk factors for PCOS. To assess the risk for PCOS associated with the FTO rs9939609 polymorphism after controlling for confounders, logistic regression analysis was performed using forward LR method to obtain adjusted OR. In the regression model, case control status was included as the dependent variable; and the main predictor (FTO rs9939609 polymorphism was categorized as AA [homozygous], AT [heterozygous] and TT [wild type]) along with confounders (all significant factors in the univariate analysis) as independent variables. To determine the risk for FTO rs9939609 polymorphism to be associated with BMI, mFG scale, serum testosterone and kisspeptin levels, forward LR was performed. This analysis categorized the FTO rs9939609 into 2 groups - mutant allele (AA and AT) and normal allele (TT) and included them as the dependent variable. Any interaction of FTO rs9939609 polymorphism with each variable was analyzed individually to test for significance. Variables found to have a significant interaction were then included to the model. Several regression models were developed for both analysis, and the best model was selected based on the goodness of fit. All analyses were performed by SPSS software (v.18.0 SPSS, Inc., Chicago, IL). The level of significance was set as 5%.

Deviations from the Hardy–Weinberg equilibrium were tested by comparison of observed and expected genotype frequencies with χ2 test. Calculation of genotype and haplotype associations for all the SNPs was carried out using SNPSTATS program (http://bioinfo.iconcologia.net/index.php?module=Snpstats). Five inheritance models (co-dominant, dominant, recessive, over-dominant and additive) were applied for statistical analysis. The best inheritance model was assessed using the Akaike information criteria (AIC) and the Bayesian information criteria (BIC) and the model with the lowest values considered the best fit.

## Results

### Risk factors for PCOS development

Results of demographic, clinical and biochemical parameters of study subjects are explained in our previous study and Table 1 summarize the major results of our previous study. Table 2 shows the association of cases of PCOS and controls based on the univariate and logistic regression analysis. PCOS is associated with BMI, serum testosterone and kisspeptin concentrations and the FTO rs9939609 polymorphism by univariate analysis. Our model showed that BMI, serum kisspeptin levels and FTO rs9939609 (AA) polymorphism remained significant after adjusting for potential confounders. The model was statistically significant with a Hosmer and Lemeshow test value of 0.34 (*p* > 0.1 taken as significant). The model also explained 24% (Nagelkerke R2) of the variance among those with PCOS and correctly classified 72% of the cases, which confirms a good fit. Meanwhile, this model demonstrated there was no significant association between genes of the HPG axis (Kiss1, GPR54, GnRH, FSHB, FSHR, LHCGR) and insulin receptor gene (INSR) with PCOS.

**Table 1:** Demographic, clinical and biochemical characteristics of the study population (results from our previous study – Branavan U et al. [28], Plos one)

	PCOS (*n* = 55)	CONTROLS (*n* = 110)	p
Age (Years)	24.67 ± 0.883	33.80 ± 0.528	0.061
BMI(Kg/m^2^)	26.89 ± 0.716	25.25 ± 0.344	0.007
mFG score	8 ± 0.445	3 ± 0.222	0.006
WC:HC	0.839 ± 0.008	0.824 ± 0.004	0.114
FBG (mg/dL)	98.81 ± 2.08	108.69 ± 2.74	0.284
Kisspeptin (nmol/L)	4.873 ± 0.238	4.127 ± 0.132	0.033
Testosterone (nmol/L)	4.713 ± 0.458	3.415 ± 0.256	0.018

*BMI* Body mass index, *mFG* modified Ferriman-Gallway score, *WC:HC* waist circumference: hip circumference, *FBG* fasting blood glucose, *TSH* Thyroid stimulating hormone, *FSH* Follicle stimulating hormone, *LH* Luteinizing hormone

**Table 2:** Risk factors for PCOS

Risk factor	Genotype	Cases *N* = 55	Controls *N* = 110	Crude OR (95% CI)	Adjusted OR (95%CI) Model ^a^
BMI (mean, SE)	–	26.89 ± 0.72	25.25 ± 0.34	1.1 (1.01–1.18)	1.1 (1.01–1.2)
Serum testosterone (mean, SE)	–	4.713 ± 0.46	3.415 ± 0.26	1.2 (1.03–1.29)	
Serum kisspeptin (mean, SD)	–	4.873 ± 0.24	4.127 ± 0.13	1.37 (1.1–1.71)	1.4 (1.14–1.85)
FTO (rs9939609) (No, %)	AA	22 (40%)	15 (13.6%)	4.9 (2.16–11.0)	5.7 (2.41–13.63)
	AT	13 (23.6%)	23 (20.9%)	2.0 (0.86–4.65)	2.2 (0.9–5.35)
	TT^R^	20 (36.3%)	72 (65.4%)	1	

^a^Logistic Regression (LR) model showing adjusted odds ratio (OR) of factors significant for PCOS

Logistic regression analysis was carried out for all the SNPs of Kiss1, GPR54, GnRH, FSHB, FSHR, LHCGR, INSR and FTO along with hormonal (kisspeptin and testosterone levels) characteristics. Only the variables with significant association are shown in the table

### Hardy–Weinberg equilibrium and mode of inheritance analysis

The genotype distributions of the FTO SNP (rs9939609) was not in the Hardy–Weinberg equilibrium in both patients and controls (*p* < 0.05).

Association between the FTO SNP (rs9939609) and PCOS risk was analyzed under five gene models (co-dominant, dominant, recessive, over-dominant and log additive).

The FTO gene rs9939609 polymorphism - under the co-dominant model, the genotypes “AA” (OR = 5.49; 95% CI -2.34-12.88; *p* < 0.05); under the dominant model genotype “A/T-A/A” (OR = 3.21 95% CI -1.62-6.37; *p* < 0.05); under recessive model genotype “A/A” (OR = 4.45, 95% CI -2.01-9.90; *p* < 0.05) and the log additive model (OR = 2.30; 95%CI -1.51-3.51; *p* < 0.05) were associated with increased risk for PCOS (Table 3).

**Table 3:** Association between FTO SNP (rs9939609) and PCOS risk under multiple models of inheritance

Model	Genotype	Controls	Cases	OR (95% CI)	p	AIC	BIC
Codominant	T/T	68 (65.4%)	20 (37%)	1.00	0.00	192.7	201.8
	A/T	23 (22.1%)	13 (24.1%)	1.92 (0.83–4.47)			
	A/A	13 (12.5%)	21 (38.9%)	5.49 (2.34–12.88)			
Dominant	T/T	68 (65.4%)	20 (37%)	1.00	0.00	195.3	201.4
	A/T-A/A	36 (34.6%)	34 (63%)	3.21 (1.62–6.37)			
Recessive	T/T-A/T	91 (87.5%)	33 (61.1%)	1.00	0.00	192.9	199
	A/A	13 (12.5%)	21 (38.9%)	4.45 (2.01–9.90)			
Overdominant	T/T-A/A	81 (77.9%)	41 (75.9%)	1.00	0.78	206.9	213
	A/T	23 (22.1%)	13 (24.1%)	1.12 (0.51–2.43)			
Logadditive	–	–	–	2.30 (1.51–3.51)	0.00	190.9	197

*OR* odds ratio, *CI* confidence interval, *AIC* Akaike information criteria, *BIC* Bayesian information criteria

The model with the lowest AIC and BIC values for a given polymorphism was considered the best-fit model. The AIC and BIC values indicated that the log additive model may serve as the best-fit model of rs9939609 polymorphism of FTO gene (Table 3).

### Association of FTO rs9939609 polymorphism with clinical and hormonal characteristics of PCOS

The best predictors of FTO rs9939609 polymorphism, with a statistically significant association were the mFG scale and serum testosterone (Table 4). Furthermore, interaction between FTO rs9939609 polymorphism with the variables (BMI, mFG, serum testosterone and kisspeptin levels) showed significant interaction between FTO AA genotype and mFG (*p* = 0.042); whereas the AT genotype of FTO gene showed a marginally significant interaction (*p* = 0.054) with mFG.

**Table 4:** Association of FTO rs9939609 polymorphism with different variables (BMI, mFG score, testosterone and kisspeptin levels

Variables	Crude OR (95% CI)	Adjusted OR (95%CI) Model 1
BMI	1.0 (0.94–1.09)	
mFG score	1.1 (1.04–1.25)	1.1 (1.02–1.23)
Testosterone	1.2 (1.05–1.32)	1.1 (1.02–1.29)
Kisspeptin	1.0 (0.82–1.22)	

### Association of FTO genotype with serum kisspeptin and testosterone levels

**Fig. 1:**
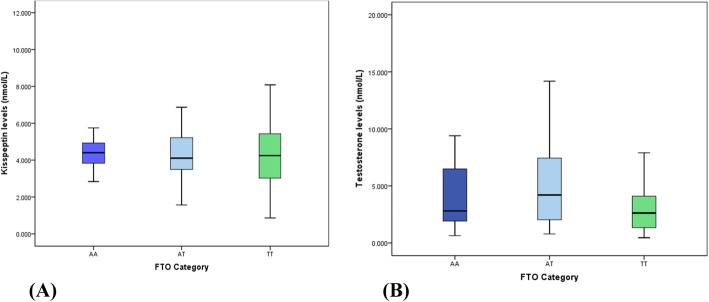
Association of FTO genotypes with serum (**a**) kisspeptin levels and (**b**) testosterone levels

Although kisspeptin levels were significantly associated with FTO genotype in the univariate analysis; the association was insignificant when adjusted for confounders (Table 4). Nevertheless, when the subjects were divided based on the FTO genotypes and compared with serum kisspeptin and testosterone levels, subjects with AA genotype had higher mean serum kisspeptin levels when compared to AT and TT genotypes (Fig. 1) while the mean testosterone concentrations were greater in subjects with the AT genotype (*p* > 0.05) (Fig. 1). In addition, serum testosterone levels were significantly higher in subjects with mutant alleles (AA + AT) when compared to those with normal allele (TT) (*p* = 0.04).

### Association of FTO genotypes with body mass index (BMI)

**Fig. 2:**
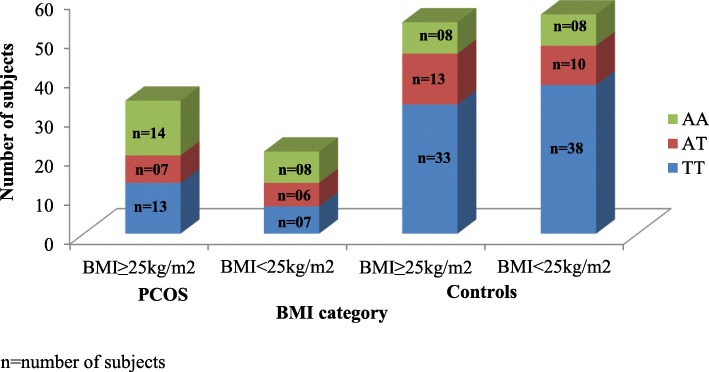
Association between FTO genotypes and BMI in cases and controls

When the subjects were subdivided based on their BMI using the Asian cut off (BMI < 25 kg/m^2^) and compared with the FTO genotype (Fig. 2) we found a significant correlation between FTO gene polymorphism and BMI (chi square value =17.05, *p* < 0.05). The frequency of AA genotype was greater among obese PCOS subjects (BMI ≥ 25 kg/m2) while the TT allele was seen in a greater proportion of controls.

### FTO with cutaneous markers of insulin resistance (Acanthosis nigricans)

**Fig. 3:**
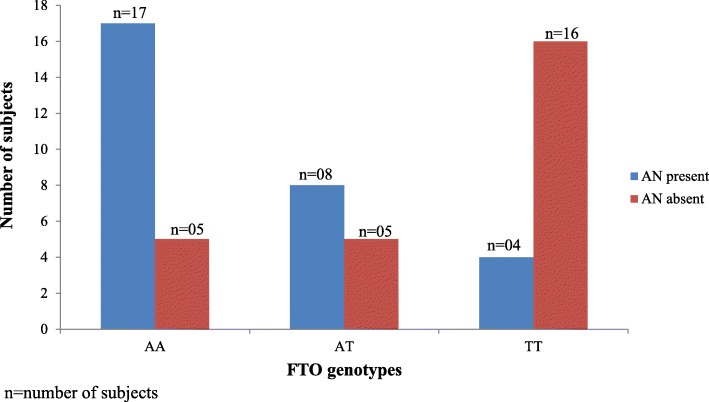
Association between FTO genotypes and Acanthosis nigricans (AN) in PCOS subjects

Acanthosis nigricans was used as a clinical marker of insulin resistance. PCOS subjects with the AA genotype had a significantly higher frequency of acanthosis nigricans when compared to those with the TT genotype (*p* < 0.05) (Fig. 3). Acanthosis was seen most commonly among women with PCOS who were obese or overweight.

## Discussion

The pathogenesis of PCOS remains complex with multiple factors deemed to determine its expression. A single cause for PCOS is unlikely [35]. Overweight and obesity affects 40–80% of women with PCOS [11, 36, 37]. Genome-wide association studies reported that obesity gene FTO has an association with PCOS, mostly among Asians [38]. Nevertheless, the mechanism underlying this association of the FTO gene polymorphism with PCOS risk remains unclear. Some researchers have reported that FTO gene influences PCOS mainly via the association with obesity or obesity-related parameters such as BMI [5, 11, 39, 40]. We found that the FTO rs9939609 variant is unequivocally associated with PCOS among a cohort of well-characterized Sri Lankan women by both logistic regression analysis and genetic inheritance analysis. While many others have shown a significant association of FTO rs9939609 polymorphism with PCOS [5, 19–21]; a few have reported the lack of such an association [11, 39, 41].

The association between insulin resistance (IR) and FTO rs9939609 polymorphism is well established. Russell et al., showed that overexpression of FTO in INS-1 pancreatic beta cells increased the first phase of insulin secretion response to glucose [42]. Interestingly, we observed that the majority of subjects with PCOS had acanthosis nigricans (a clinical marker associated with IR - thickened, velvety, relatively darker areas of skin on the neck, armpit and in skin folds [43]) and had the A allele (either AT or AA) in the FTO rs9939609 polymorphism (Fig. 3). Therefore, it is reasonable to conclude that IR is associated with FTO rs9939609 polymorphism among Sri Lankan women with well characterized PCOS.

No studies have been reported so far on a possible association between serum kisspeptin levels and the FTO gene polymorphism in PCOS. Although we found kisspeptin to be significantly associated with FTO genotype in the univariate analysis; the association became insignificant when adjusted for confounders (Table 4). Nevertheless, we found that subjects with the FTO gene mutant alleles (AT and AA) had higher serum kisspeptin levels when compared to subjects with the normal allele (*p* > 0.05) (Fig. 1). The basis for an association of FTO gene polymorphism with increased serum kisspeptin levels is ill understood. However, association between FTO gene rs9930609 polymorphism and serum leptin levels has been investigated by several groups. Rutters et al. found FTO A allele to be associated with higher BMI and leptin levels at the age of 12, with the association being stronger at the age of 17 [44]. Magno et al. showed that those with the AA genotype had higher values of leptin than those with TT and AT [45]. Yet others have reported the expression of Kiss1 gene (gene that produces kisspeptin) may be induced by leptin [46, 47]. Smith et al., 2006 concluded Kiss1 neurons are direct targets for regulation by leptin and suggested that the reproductive deficits associated with leptin-deficient states may be attributable, in part, to diminished expression of the Kiss1 gene [48]. Therefore, we propose that the FTO gene polymorphism exerts an effect on the Kiss1 gene (that produces kisspeptin) through leptin, which leads to increased serum kisspeptin levels. Hence, further evaluation of any association between FTO gene variant and kisspeptins level is warranted. Such an approach to investigating the Kiss-1 system and associated biochemical pathways may help define therapeutic interventions that could target the reproductive consequences of PCOS.

Recent studies have examined the relationship of the FTO gene with other PCOS-associated-phenotypes, including obesity, glucose intolerance, and insulin resistance [23, 49]. However, the association between the FTO gene and hyperandrogenemia remains equivocal. In a study of women from a UK population, no relationship between FTO genotype and androgen levels was observed [5]. Moreover, in a Polish population, there was no difference in testosterone, SHBG, and free androgen index values according to genotype [49]. However, a study of 288 European women with PCOS found the rs9939609 variant of the FTO gene to be associated with hyperandrogenemia [11]. Our study clearly demonstrates, using forward LR analysis, that the rs9939609 variant of the FTO gene is significantly associated with the mFG score for hirsutism (OR = 1.1, 95%CI = 1.02–1.23, *p* < 0.05) and serum testosterone levels (OR = 1.1, 95%CI = 1.02–1.29, *p* < 0.05) that remained significant after controlling for confounders (Table 3). Hence, we propose that the FTO gene rs9939609 variant is indeed associated with hyperandrogenemia among Sri Lankan women with the well characterized phenotype of PCOS that manifests from adolescence. However, further studies in larger samples is required to confirm our findings.

This research being clinically based led to some inevitable drawbacks. These drawbacks were deviations from the original design, viz. in recruitment of subjects and data collection. The cases and control were not strictly age matched, where the mean age of controls is higher than that of the affected cohort of women with PCOS. Although this may have been due to chance, it is unlikely to have confounded the calculated risk, as age is usually not associated as a confounder of genetic testing. The explanation for the marginally older cohort of controls is that the volunteer women were selected from a single large work setting; where the controls required to be confirmed as to have regular cycles over a long period of time extending to their early adult life and had even achieved fertility; all of which clearly ensured the non-inclusion of the milder phenotype of PCOS into the control group.

Furthermore, due to the limitation of funding, LH, FSH hormones levels and thyroid testing were not determined in the control subjects. In addition, AMH level was not measured in neither cases nor controls. Hence the gonadotropin, thyroid hormones, AMH and their association with genetic polymorphisms could not be compared between cases and control subjects.

Complete data sets of the routine clinic based metabolic parameters such as lipid profile, liver enzymes and uric acid were not accessible from clinic notes in all women. Tracing through the current hospital laboratory management systems proved unsuccessful in some. Hence the association of the clinical manifestation of the metabolic syndrome with the genetic polymorphisms of PCOS could not be compared between cases and controls.

## Conclusion

We conclude that the rs9939609 variant of FTO gene is significantly associated with hyperandrogenemia, acanthosis nigricans and the BMI among young Sri Lankan women with the well characterized phenotype manifesting from adolescence. Meanwhile serum kisspeptin levels have no significant association with the FTO genotypes when adjusted for confounders.

## Data Availability

The datasets generated and/or analyzed during the current study are available in the Figshare repository (https://figshare.com/articles/Complete_Gene_results_BMC_sav/11637264).

## Abbreviations

AIC: Akaike information criteria
AMH: Anti mullerian hormone
AN: Acanthosis nigricans
BIC: Bayesian information criteria
BMI: Body mass index
CI: Confidence interval
FSH: Follicle stimulating hormone
FSHB: Follicle stimulating hormone beta subunit
FSHR: Follicle stimulating hormone receptor
FTO: Fat mass and obesity associated gene
GnRH: Gonadotropin releasing hormone
HPG: Hypothalamic pituitary axis
INSR: Insulin receptor
IR: Insulin resistance
LH: Luteinizing hormone
LHB: Luteinizing hormone beta subunit
LHCGR: Luteinizing hormone/choriogonadotropin receptor
LR: Logistic regression
mFG: Modified Ferriman–Gallwey
OR: Odd ratio
PCOS: Polycystic ovary syndrome
SHBG: Sex hormone binding globulin
SNP: Single nucleotide polymorphism
